# Anticancer regimens containing third generation taxanes SB-T-121605 and SB-T-121606 are highly effective in resistant ovarian carcinoma model

**DOI:** 10.3389/fphar.2022.971905

**Published:** 2022-11-09

**Authors:** Karolina Seborova, Kamila Koucka, Alzbeta Spalenkova, Petr Holy, Marie Ehrlichova, Tomas Sychra, Lei Chen, Hersh Bendale, Iwao Ojima, Cristian Sandoval-Acuña, Jaroslav Truksa, Pavel Soucek, Radka Vaclavikova

**Affiliations:** ^1^ Toxicogenomics Unit, National Institute of Public Health, Prague, Czech Republic; ^2^ Laboratory of Pharmacogenomics, Biomedical Center, Faculty of Medicine in Pilsen, Charles University, Pilsen, Czech Republic; ^3^ Third Faculty of Medicine, Charles University, Prague, Czech Republic; ^4^ Department of Surgery, University Hospital Kralovske Vinohrady and Third Faculty of Medicine, Charles University, Prague, Czech Republic; ^5^ Institute of Chemical Biology & Drug Discovery, State University of New York at Stony Brook, Stony Brook, NY, United States; ^6^ Institute of Biotechnology of the Czech Academy of Sciences, BIOCEV Research Center, Vestec, Czech Republic

**Keywords:** ovarian carcinoma, resistance, paclitaxel, SB-T taxanes, efficacy, *in vitro*, *in vivo*

## Abstract

Taxanes are widely used in the treatment of ovarian carcinomas. One of the main problems with conventional taxanes is the risk of development of multidrug resistance. New-generation synthetic experimental taxoids (Stony Brook Taxanes; SB-T) have shown promising effects against various resistant tumor models. The aim of our study was to compare the *in vitro* efficacy, intracellular content, and *in vivo* antitumor effect of clinically used paclitaxel (PTX) and SB-Ts from the previously tested second (SB-T-1214, SB-T-1216) and the newly synthesized third (SB-T-121402, SB-T-121605, and SB-T-121606) generation in PTX resistant ovarian carcinoma cells NCI/ADR-RES. The efficacy of the new SB-Ts was up to 50-times higher compared to PTX in NCI/ADR-RES cells *in vitro*. SB-T-121605 and SB-T-121606 induced cell cycle arrest in the G2/M phase much more effectively and their intracellular content was 10–15-times higher, when compared to PTX. Incorporation of SB-T-121605 and SB-T-121606 into therapeutic regimens containing PTX were effective in suppressing tumor growth *in vivo* in NCI/ADR-RES based mice xenografts at small doses (≤3 mg/kg), where their adverse effects were eliminated. In conclusion, new SB-T-121605 and SB-T-121606 analogs are promising candidates for the next phase of preclinical testing of their combination therapy with conventional taxanes in resistant ovarian carcinomas.

## 1 Introduction

Conventional taxanes paclitaxel (PTX, Taxol^®^) and docetaxel (Taxotere^®^) show high antitumor efficacy and belong to the most widely used chemotherapeutics. PTX is the gold standard therapeutic drug in a combination regimen with platinum derivatives for first-line treatment of ovarian carcinoma. New therapeutic approaches including poly (ADP-ribose) polymerase inhibitors (PARPi) were approved as frontline maintenance treatment for all patients with epithelial ovarian cancer (EOC) who respond to platinum-based chemotherapy and for BRCA-associated ovarian cancer (Moore and Pothuri, 2021). Among taxanes, PTX formulated as albumin-bound nanoparticles (Abraxane^®^), has recently become an option for treatment of pancreatic carcinoma, a cancer type generally associated with very poor prognosis partly due to drug resistance (Lemstrova et al., 2016). At present, Abraxane^®^ is used also for therapy of triple negative breast cancer (van Veldhuisen et al., 2019; Malhotra and Emens, 2020).

Intrinsic and acquired resistance of tumor cells limits clinical use of conventional taxanes. Development of taxane resistance is a very complex phenomenon driven by a number of mechanisms, including qualitative and quantitative alterations of microtubules and tubulin proteins, certain drug membrane transporters, and changes in the levels of cell cycle-related proteins and chemokine signal transduction proteins (Kavallaris, 2010; Maloney et al., 2020; Das et al., 2021). Due to the large number of patients with distinct phenotypes that do not optimally respond to taxanes, a search for new therapeutic agents combatting these resistant tumors is currently needed.

**FIGURE 1:**
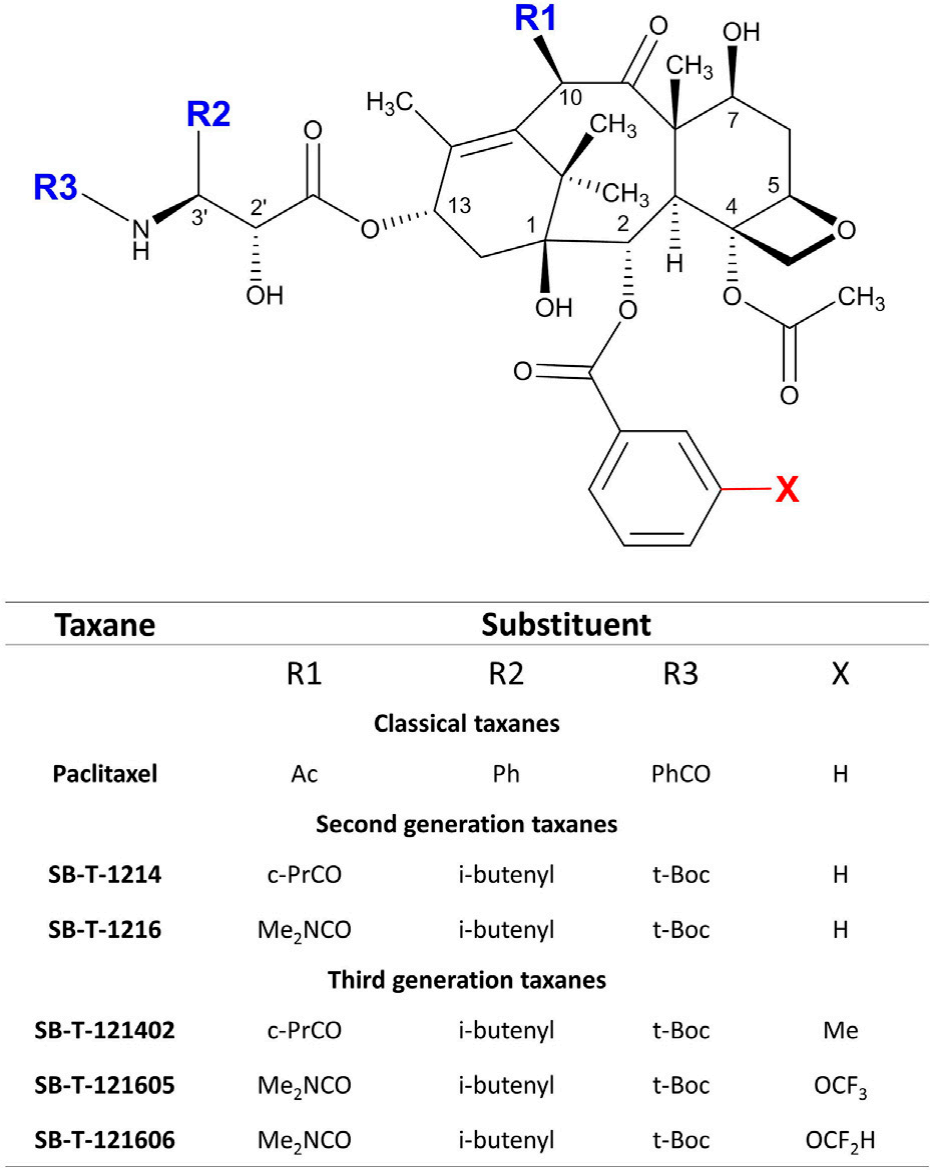
Scheme of structure modifications in second and third generation of SB-Ts in comparison to conventional PTX.

Using extensive structure-activity relationship (SAR) studies of taxanes, the laboratory of Prof. I. Ojima developed second and third generation taxoids with synthetic modifications at the C-2, C-10, and C-3′ positions of PTX (Figure 1) (Ojima and Das, 2009; Seitz et al., 2015, 2018) called Stony Brook Taxanes (SB-Ts). These modifications of the PTX structure produced molecules that overcome PTX resistance and reduce drug efflux by P-glycoprotein and metabolic deactivation by cytochrome P450 (CYP) enzymes. In fact, some of the second generation SB-Ts have already demonstrated effectiveness in various cancer cell models *in vitro* and their mechanisms of action have been described (Vobořilová et al., 2011; Ehrlichová et al., 2012; Jelínek et al., 2015; Wang et al., 2020). *In vivo*, SB-T-1214, SB-T-12854, and IDN5109 analogs suppressed spontaneous rat lymphoma growth more effectively than PTX (Otová et al., 2012) and in mouse PaCa-44 cell line xenografts, SB-T-1216 suppressed the Hedgehog pathway overexpression seen in tumors from patients with pancreatic ductal carcinoma (Mohelnikova-Duchonova et al., 2017). Despite a substantial increase in antitumor efficacy, a high toxicity profile of the second generation of SB-Ts was observed in the examined mouse models, limiting the enthusiasm for their further utilization. However, combined regimens of conventional taxanes with small doses of SB-Ts could potentially be effective and associated with lower toxicity at the same time. Furthermore, a new third generation of SB-Ts have been designed and synthesized with the aim of overcoming the toxicity issues of the second generation. Mechanisms of *in vitro* efficacy and *in vivo* effect of the third generation taxanes have not been deeply investigated to date.

In this study, we focused our attention on the comparison of the effects of second generation SB-Ts (SB-T-1214 and SB-T-1216) and their corresponding C-2 derivatives of the third generation (SB-T-121402, SB-T-121605, and SB-T-121606) in the PTX resistant ovarian carcinoma model NCI/ADR-RES both *in vitro* and *in vivo*. We particularly followed 1) the antitumor efficacy, cell cycle changes, and intracellular content of both SB-T generations and PTX *in vitro* and 2) the *in vivo* efficacy of the most *in vitro* effective third generation SB-Ts in comparison to PTX alone or in combination regimens. We provide for the first time an overview of the efficacy of combined regimens based on conventional and experimental taxanes that may be further considered in translational research on therapy of resistant tumors.

## 2 Materials and methods

### 2.1 Materials

Taxanes of the second (SB-T-1214, SB-T-1216) and third generation (SB-T-121402, SB-T-121605, and SB-T-121606) were developed and synthesized at the Institute of Chemical Biology & Drug Discovery (Stony Brook University, Stony Brook, NY, United States) (Ojima et al., 2008; Wang et al., 2020) (Figure 1). The design and synthesis of new generation taxoids is described elsewhere (Ojima and Das, 2009; Seitz et al., 2015, 2018). PTX for *in vitro* experiments was obtained from Sigma Aldrich (St. Louis, MA, United States). Taxanes were dissolved in DMSO for stock/working solution before use, the concentration of stock of each taxane was 10 mM. Infusion solution of PTX for *in vivo* experiments - Paclitaxel EBEWE 6 mg/ml came from Ebewe Pharma (Unterach am Attersee, Austria).

### 2.2 Cell line and culture conditions

Human ovarian carcinoma cell line NCI/ADR-RES as a model for the study of multi-drug resistance was obtained from the National Cancer Institute, Frederick, MD, United States. This cell line was grown in RPMI 1640 medium with L-glutamine (300 mg/L), NaHCO_3_ (2.0 g/L), penicillin (100 U/ml), streptomycin (100 μg/ml), sodium pyruvate (1 mM), HEPES buffer (15 mM), and fetal bovine serum at a final concentration of 10% (PAN-Biotech, Aidenbach, Germany). The cell line was cultured in an incubator at 37°C in a humidified atmosphere with 5% CO_2_. *Mycoplasma* contamination was routinely tested using the MycoAlert *mycoplasma* detection kit (Lonza, Basel, Switzerland). Authenticity of the NCI/ADR-RES cell line was confirmed by the short tandem repeats (STR) DNA profiling analysis. STR loci were amplified using PowerPlex® 16 System (Promega, Madison, WI, United States) according to the manufacturer’s instructions. Electrophoretic analysis was performed using A 3130 xl Genetic Analyzer and the data were analyzed with GeneMapper^®^ID-X (both Applied Biosystems, Waltham, MA, United States).

### 2.3 Cell growth and survival

Cells were seeded in concentrations of 5 × 10^3^ cells per well in a 96-well plate and cultured in a complete growth medium. After 18 h, the growth medium was replaced with fresh medium alone (control) or containing PTX (10–100,000 nM), second generation (1–10,000 nM), or third generation (0.3–3 000 nM) SB-Ts. The total time of incubation with taxanes was 72 h. Cell viability was then measured fluorometrically using the CellTiter-Blue® viability assay (Promega) in an Infinite M200 plate reader (Tecan, Männedorf, Switzerland) according to manufacturers’ instructions. All experiments were repeated three times.

An alternative approach to measure cell growth and death was performed by real time monitoring of cells by the Etaluma LS720 microscope (Etaluma, Carlsbad, CA, United States). Cells were seeded at 4 × 10^3^ per well in a 96-well plate, left undisturbed overnight in order to attach to the surface and the tested compounds were added the next day. A picture of each well was taken every 3 h over a period of 96 h. Detection of dead cells was performed using the fluorescent dye SYTOX Green (ThermoFisher Scientific, Waltham, MA, United States) with excitation and emission wavelengths of 483 nm and 503 nm, respectively. Analysis of cell proliferation and cell death was performed using the Lumaquant 8.1 software (Etaluma) and expressed as cellular confluence and number of dead cells over time, subtracting dead cells at the start of the experiment after addition of the compounds, thus gaining 0 dead cells at time 0.

### 2.4 Analysis of cell cycle

For cell cycle analysis, cells were seeded in concentrations of 5 × 10^5^ cells per well into a 6-well plate in the standard growth medium. Cells were allowed to attach to the surface for 18 h and after that, the culture medium was replaced by a fresh medium containing SB-Ts (concentration range: 20–300 nM) or PTX (300–6000 nM). Control cells were incubated with medium without taxanes. After 24 h of incubation, cells were harvested, washed twice with PBS, and fixed in cold 70% ethanol at 4°C overnight. Fixed cells were washed twice with PBS and stained by propidium iodide solution (50 μg/ml propidium iodide and 10 μg/ml RNase) in ultrapure water for 10 min. Fluorescence was measured by the flow cytometer BD FACSVerse (Becton Dickinson, Franklin Lakes, NJ, United States). For each sample, 20,000 single cell events were recorded and data were analyzed using the BD FACSuite software (Becton Dickinson). All measurements were performed in independent biological duplicates.

### 2.5 Assessment of intracellular content of taxanes

For the uptake assay, 5 × 10^5^ of cells in a 24-well plate were either exposed to a fresh medium or the medium containing 10 μM PTX or SB-Ts (SB-T-1216, SB-T-121605, or SB-T-121606). After 120 min of incubation, the medium containing the taxane was quickly aspirated and adherent cells were washed three times with ice-cold PBS and harvested by trypsinization. Sodium dodecyl sulfate was added up to 2% final concentration for lysis of cells and release of the drug. Taxanes were extracted by 2 × 3.5 ml of ethyl acetate (vortexed three times for 15 s). The test tubes were centrifuged (1 500 × *g* for 10 min at RT) and clear ethyl acetate extracts (6 ml each) were evaporated to dryness under a mild nitrogen stream. Intracellular content of taxanes was measured using the HPLC HP1100 machine with autosampler (Agilent Technologies, Santa Clara, CA, United States) as described previously in detail (Václavíková et al., 2003; Ehrlichova et al., 2005). The dry extracts were dissolved in 200 μL of the mobile phase methanol:water, 70:30 [v/v]. The following HPLC conditions were used: 100 μL sample loop, Macherey-Nagel column 4 × 250 mm with Nucleosil 100–5 C18, flow 1.0 ml/min, detection at 230 nm. Chromatograms were analyzed by DataApex Clarity^TM^ version 8.0 (DataApex, spol. s.r.o., Prague, Czech Republic). PTX, SB-T-1216, SB-T-121605, or SB-T-121606 (1 μM), added to 1 ml of pure RPMI 1640 medium with 10% FBS, served as external standards and were processed in the same way as the samples.

### 2.6 Tumor xenografts

Tumor xenograft experiments involving mouse models were approved by the Ministry of Agriculture of the Czech Republic (approval no. 9806/2018MZE-17214), the Ethical Committee of the National Institute of Public Health in Prague, and the Committee of the Ministry of Health of the Czech Republic for the Project of Experiments (approval no. MZDR20330/2018-4/OVZ from 18 May 2018). Female athymic Nude Crl: NU(NCr)-Foxn1^nu^ mice aged 4–6 weeks were obtained from Charles River Laboratories (Freiburg, Germany) and housed in pathogen-free environment. 200 µL cell suspension in PBS with 2 × 10^6^ NCI/ADR-RES cells was injected subcutaneously into the dorsal flanks of each mice. Application of taxanes was initiated after tumors reached a size of ∼ 100 mm^3^. In total, 65 xenografts were prepared and divided into groups consisting of five mice: 1/untreated control group, 2/group treated with 10 mg/kg of PTX, 3/combination of 9 mg/kg of PTX with 1 mg/kg of SB-T-121605, 4/combination of 7 mg/kg of PTX with 3 mg/kg of SB-T-121605, 5/combination of 5 mg/kg of PTX with 5 mg/kg SB-T-121605, 6/combination of 9 mg/kg of PTX with 1 mg/kg of SB-T-121606, 7/combination of 7 mg/kg of PTX with 3 mg/kg of SB-T-121606. To completely distinguish efficacy of a PTX and SB-T combination from SB-T alone, we additionally performed experiment with groups of mice (five mice in each group) with regimens consisting of 1 mg/kg or 3 mg/kg SB-T-121605 and SB-T-121606 and compared them with the control group.

Each group received intraperitoneally two doses per week of PTX or SB-T-121605/SB-T-121606 or a combination of PTX with SB-Ts, in a total volume of 200 µL. Control groups received 200 µL of vehicle (4.5% DMSO in sterile water for tissue culture) (PAN-Biotech). Tumor size was measured by digital calipers in weekly intervals and tumor volume was calculated in mm^3^ by the standard formula (W^2^×L)/2, where L (length) and W (width) are measured as major diameters of the tumor in millimeters. Animals were sacrificed after 7 cycles of treatment, or based on their physical condition during the treatment.

### 2.7 Statistics

IC_50_ values for each taxane in the *in vitro* study were calculated by the GraphPad Prism 6.0 software (GraphPad Software, San Diego, CA, United States). Values are displayed as mean ± S.D. of three independent experiments. The percentages of taxane uptake by the cells are expressed as mean ± S.D. of three separate determinations. *In vivo* comparison of tumor growth and weight in individual groups of xenografts was analyzed by the two-tailed Student’s t-test. *p*-values <0.05 were considered statistically significant.

## 3 Results

### 3.1 Sensitivity of NCI/ADR-RES cells to PTX and SB-Ts *in vitro*

**FIGURE 2:**
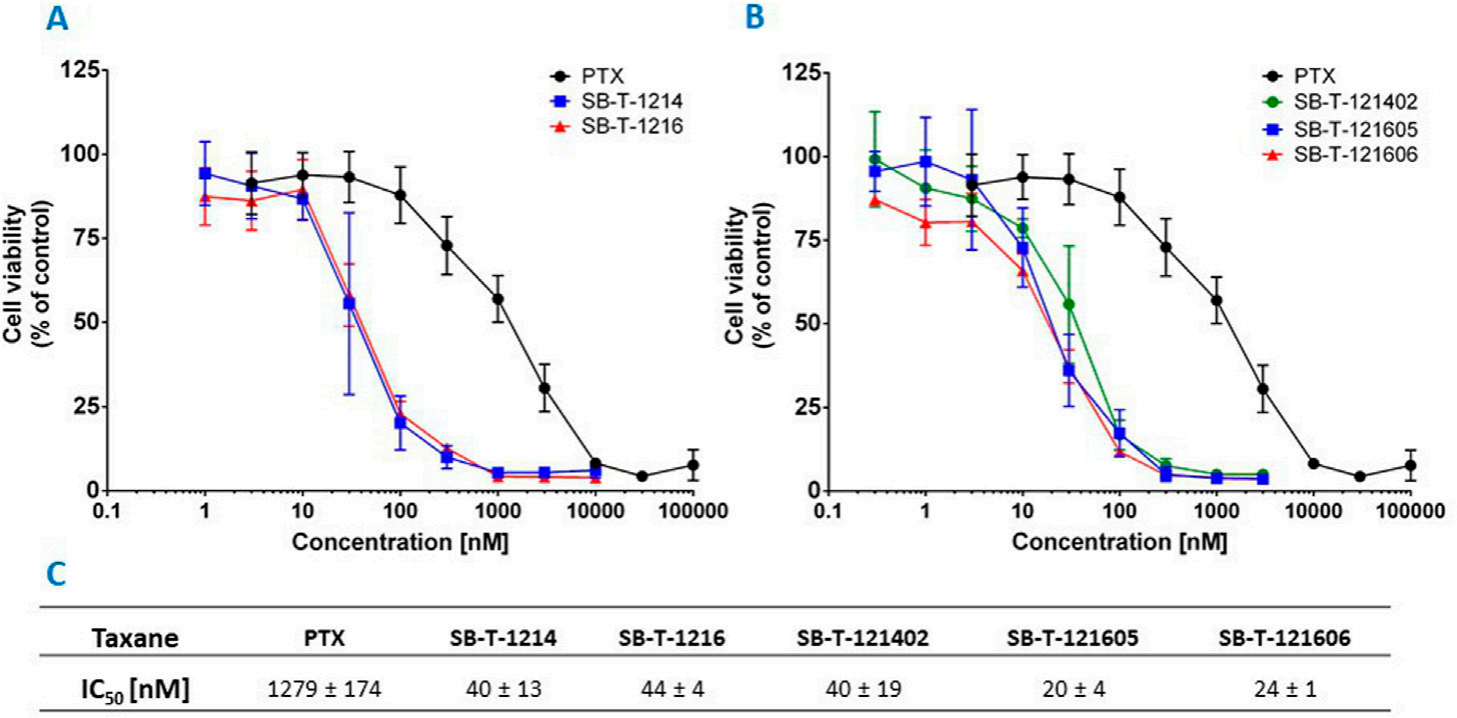
Graph representing the cell viability (% of control cells) of NCI/ADR-RES cells treated with PTX or **(A)** second generation or **(B)** third generation SB-Ts for 72 h. **(C)** Table of IC_50_ values for studied taxanes. Mean ± S.D. of three independent experiments displayed.

The efficacy of PTX and SB-Ts was analyzed *via* determination of IC_50_ value for each taxane after 72 h of incubation with NCI/ADR-RES cells. Compared to PTX, the effect of second and third generation SB-Ts on the survival of cells was markedly higher. NCI/ADR-RES cells were highly resistant to PTX with IC_50_ 1279 ± 174 nM, while the second generation SB-T-1214 and SB-T-1216 were strikingly more effective (40 and 44 nM, respectively) (Figure 2). Regarding the third generation, SB-T-121402 had similar efficacy as both taxanes of the second generation SB-Ts (40 nM). However, when analyzing the results of the “1214” branch (SB-T-1214 and SB-T-121402), higher variability between individual experiments compared to the “1216” branch was found, suggesting lower stability of the “1214″ branch. The third generation SB-Ts of the “1216” branch (SB-T-121605 and SB-T-121606) were the most effective of all tested compounds with IC_50_ values of 20 and 24 nM, respectively, i.e. about twice as effective as second generation SB-Ts and more than 50-fold stronger than PTX.

**FIGURE 3:**
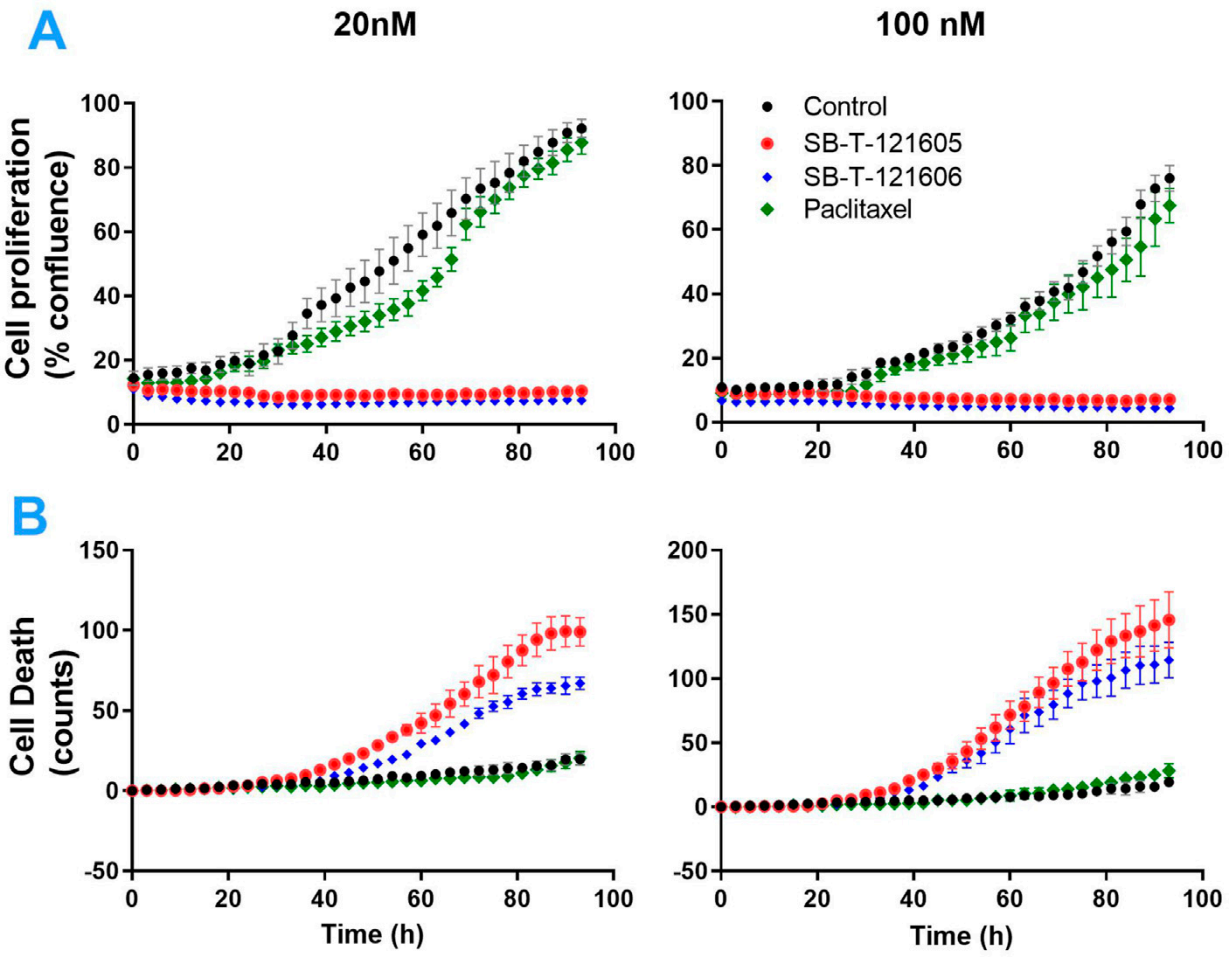
Proliferation **(A)** and death **(B)** of NCI/ADR-RES cells measured by the real-time monitoring for 96 h without taxane treatment (black curves) and after the treatment with 20 nM and 100 nM PTX (green curves), SB-T-121605 (red curves), and SB-T-121606 (blue curves). Data are present as means ± SD of three independent measurements.

**FIGURE 4:**
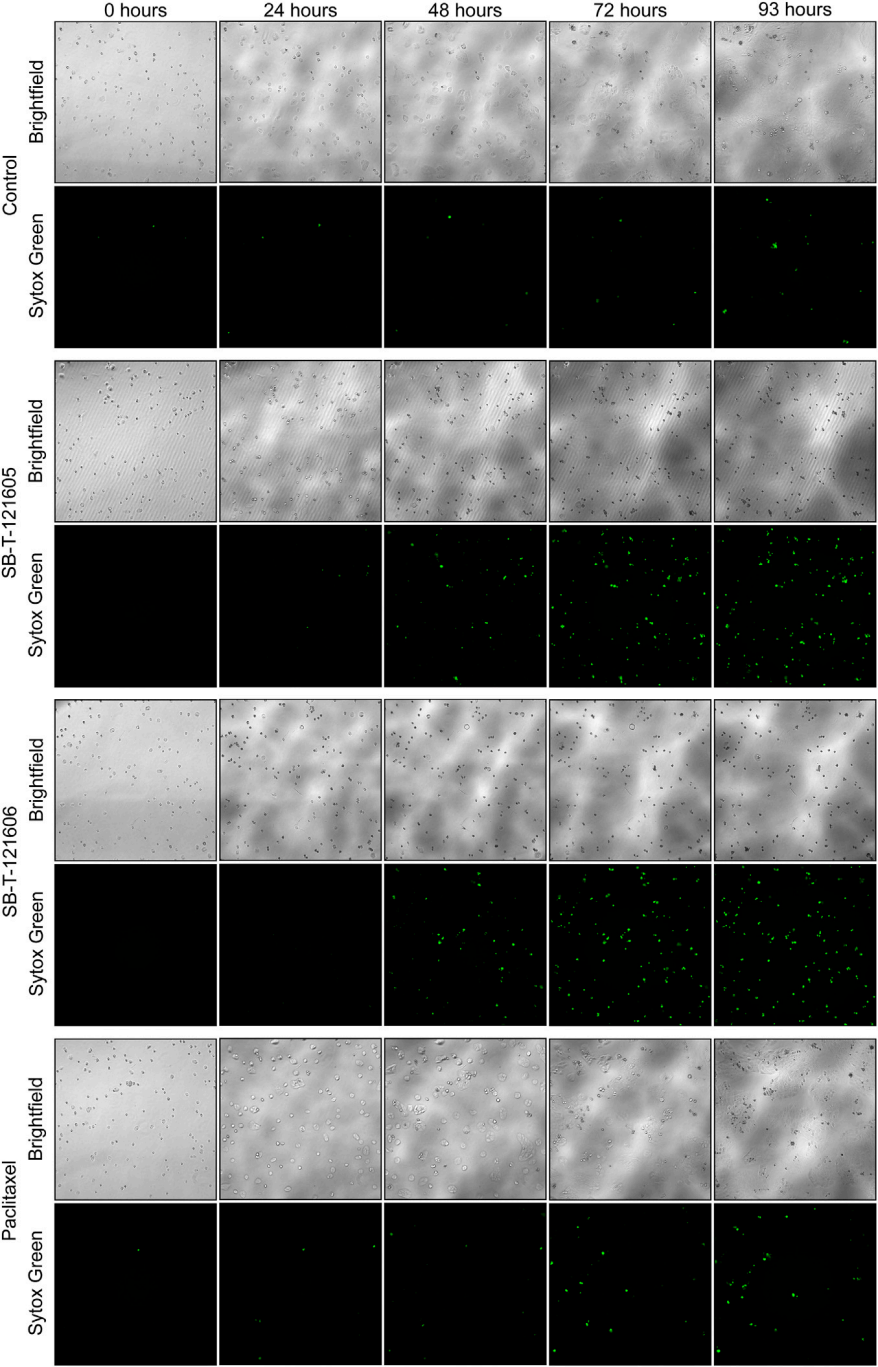
Etaluma LS720 microscope images of NCI/ADR-RES control cells and cells treated by 20 nM Paclitaxel, SB-T-121605 or SB-T-121606 for 93 h. For each time and condition, the image of native cells (Brightfield) and cells with fluorescent dye Sytox Green was taken.

An independent validation of the results obtained by the CellTiter-Blue^®^ viability assay was carried out using the real time setting by the Etaluma720 high-throughput microscope. It clearly showed that the resistance of NCI/ADR-RES cells to PTX is due to the lack of its effect on both their proliferation and cell death stimulation. In contrast, third generation SB-Ts clearly halted cellular proliferation even at low (20 nM) concentrations and they also induced profound death of the NCI/ADR-RES cell line. SB-T-121605 was in this regard slightly more potent compared to SB-T-121606 as shown in Figure 3. These data thus complement the above findings based on IC_50_ values. No immediate changes were observed at the beginning of the incubation, cells were rounded. After 24 h of incubation, viable cells started to look less rounded and became rough as judged by microscopic evaluation. After 48 h, the cells started forming colonies. In the control group of cells, fluorescence staining showed signs of cell death at the last time point (93 h). Treatment with both SB-Ts (SB-T-121605 and SB-T121606) at the 20 nM/100 nM concentration induced rapid and widespread cell death especially at the 48 h’ time point, evidenced by rounding and smoothing of the attached cells, which also became smaller. PTX treatment, on the other hand, had almost no effect on proliferation or death of NCI/ADR-RES cells. No other morphological changes were observed and the results were similar for both tested concentrations of SB-Ts (Figure 4). The above findings led to the selection of the third generation SB-T-121605 and SB-T-121606 for subsequent intracellular content assessment and *in vivo* study.

### 3.2 Cell cycle analysis

**FIGURE 5:**
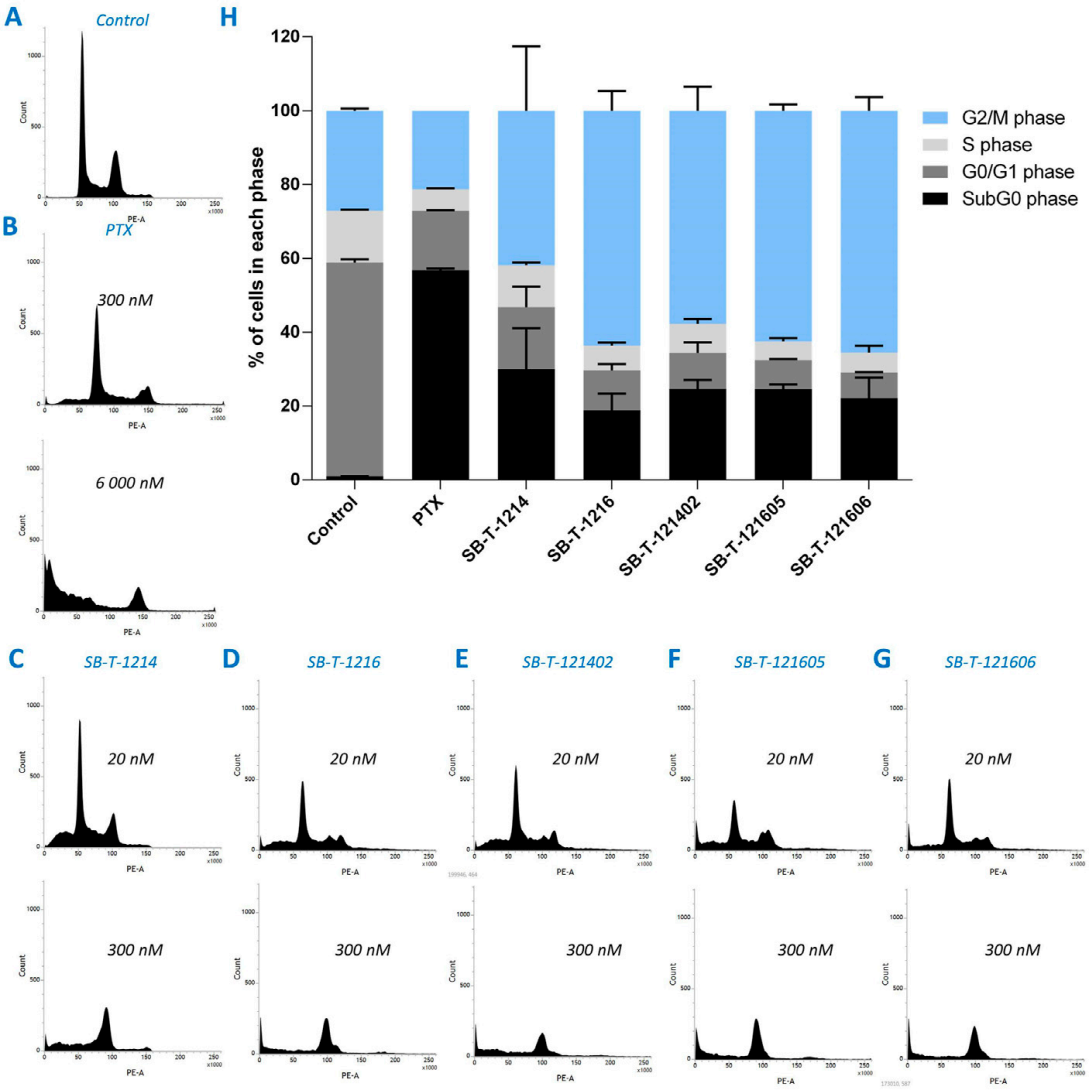
Visualization of the distribution of cell cycle phases in NCI/ADR-RES cells incubated with pure medium **(A)**, PTX **(B)**, and different SB-Ts at 20 or 300 nM concentration **(C–G)**. Cell cycle analysis **(H)** shows percentage of cells in different cell cycle phases for 6000 nM PTX and 300 nM SB-Ts. Error bars present standard deviation of two individual experiments.

Cell cycle changes were analyzed by flow cytometry after 24 h of incubation of NCI/ADR-RES cells with PTX or different SB-Ts. Incubation with 300 nM PTX (Figure 5B) did not cause significant changes in the cell cycle distribution compared to the control (Figure 5A). Incubation with 6000 nM PTX (Figure 5B) showed a significant G2/M block and arresting of most of the cells in the subG0 (apoptotic) phase. Similarly to PTX, second (SB-T-1214 and SB-T-1216) and third (SB-T-121402, SB-T-121605, and SB-T-121606) generation SB-Ts produced a G2/M block (Figures 5C–G), although at a much lower concentration (300 nM) than PTX (6000 nM) and the proportion of cells in the sub-G0 phase was much lower when compared to PTX. NCI/ADR-RES cells incubated with 20 nM SB-Ts showed only small differences compared to control cells besides a larger sub-G0 population fraction in treated cells (Figures 5C–G). At the 300 nM concentration, all SB-Ts uniformly produced a G2/M block (Figures 5C–G). The G2/M peak in cells incubated with SB-T-121605 was slightly higher compared to other SB-Ts. Taken together, SB-T-1216 and all three third generation SB-Ts showed very similar effects on the cell cycle (Figure 5H). In accordance with the IC_50_ results, SB-T-1214 exerted higher variability among individual experiments, supporting its lower stability.

### 3.3 Intracellular content of PTX, SB-T-1216, SB-T-121605, and SB-T-121606 in resistant ovarian cancer cells *in vitro*

**FIGURE 6:**
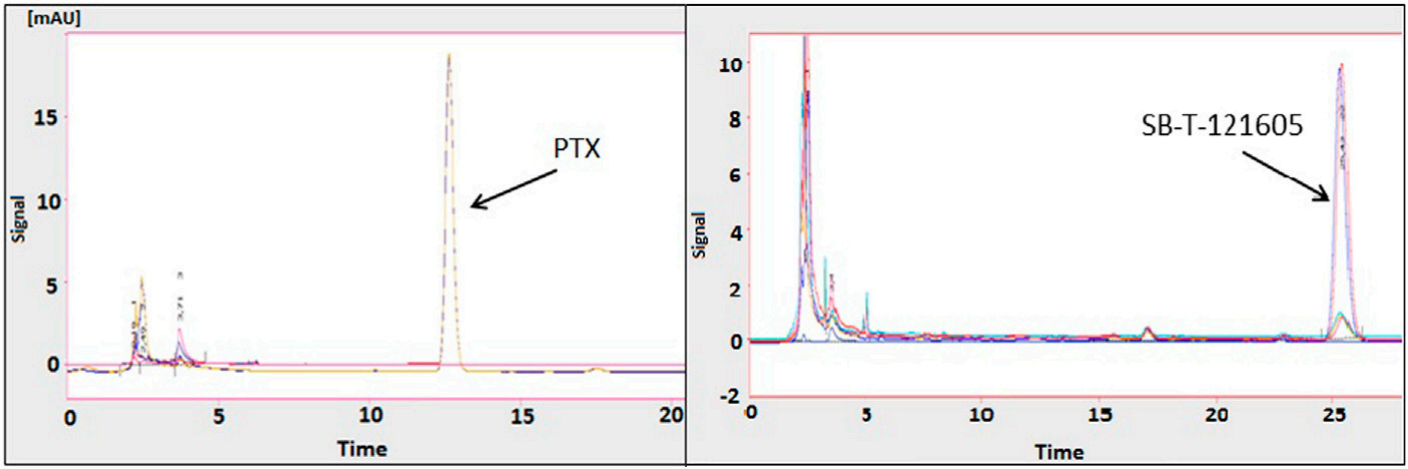
Typical chromatograms of PTX and SB-T-121605 in NCI/ADR-RES cells. For details, see the Materials and Methods section.

Intracellular concentrations of the second generation SB-T-1216 and the most promising third generation SB-T-121605 and SB-T-121606 were estimated and compared with PTX in highly resistant NCI/ADR-RES ovarian cancer cells. The intracellular content was initially determined in cells exposed to 10 μM PTX, SB-T-1216, SB-T-121605, and SB-T-121606 with analysis by HPLC. The amounts of intracellular PTX and SB-Ts after 120 min incubation are shown in Table 1. NCI/ADR-RES cells showed 6–15.5-fold higher levels of SB-Ts in comparison to PTX. The differences in the intracellular content of SB-Ts were significant as shown in Figure 6. SB-T-121605 was accumulated 15.5-fold more effectively than PTX and had the highest concentration among the examined taxane derivatives. The comparison of chromatograms for PTX and SB-T-121605 content is shown directly in Figure 6.

**TABLE 1:** Comparison of proportions of taxanes absorbed into NCI/ADR-RES ovarian cancer cells after 120 min incubation with 10 µM PTX, SB-T-1216, SB-T-121605, and SB-T-121606 taxanes and their HPLC detection. Significant results are marked as: *** (*p* ≤ 0.001).

Taxane	Percentage of absorbed drugs in NCI/ADR-RES cells^a^	Significance of differences between SB-T and PTX absorption
Conventional taxane		
PTX	0.6 ± 0.3	---
Second-generation taxane		
SB-T-1216	3.6 ± 0.3	3.52E-04***
Third-generation taxane		
SB-T-121605	9.3 ± 0.8	9.32E-04***
SB-T-121606	6.2 ± 0.5	1.18E-04***

^a^ The percentage of taxanes absorbed by the cells (0.5 × 10^6^/ml/well) after 120 min incubation and HPLC, detection is expressed as mean (%) ± S.D., of three separate experimental values (n = 3).

### 3.4 *In vivo* efficacy of the third generation taxoid in individual regimen and in combination with PTX

**FIGURE 7:**
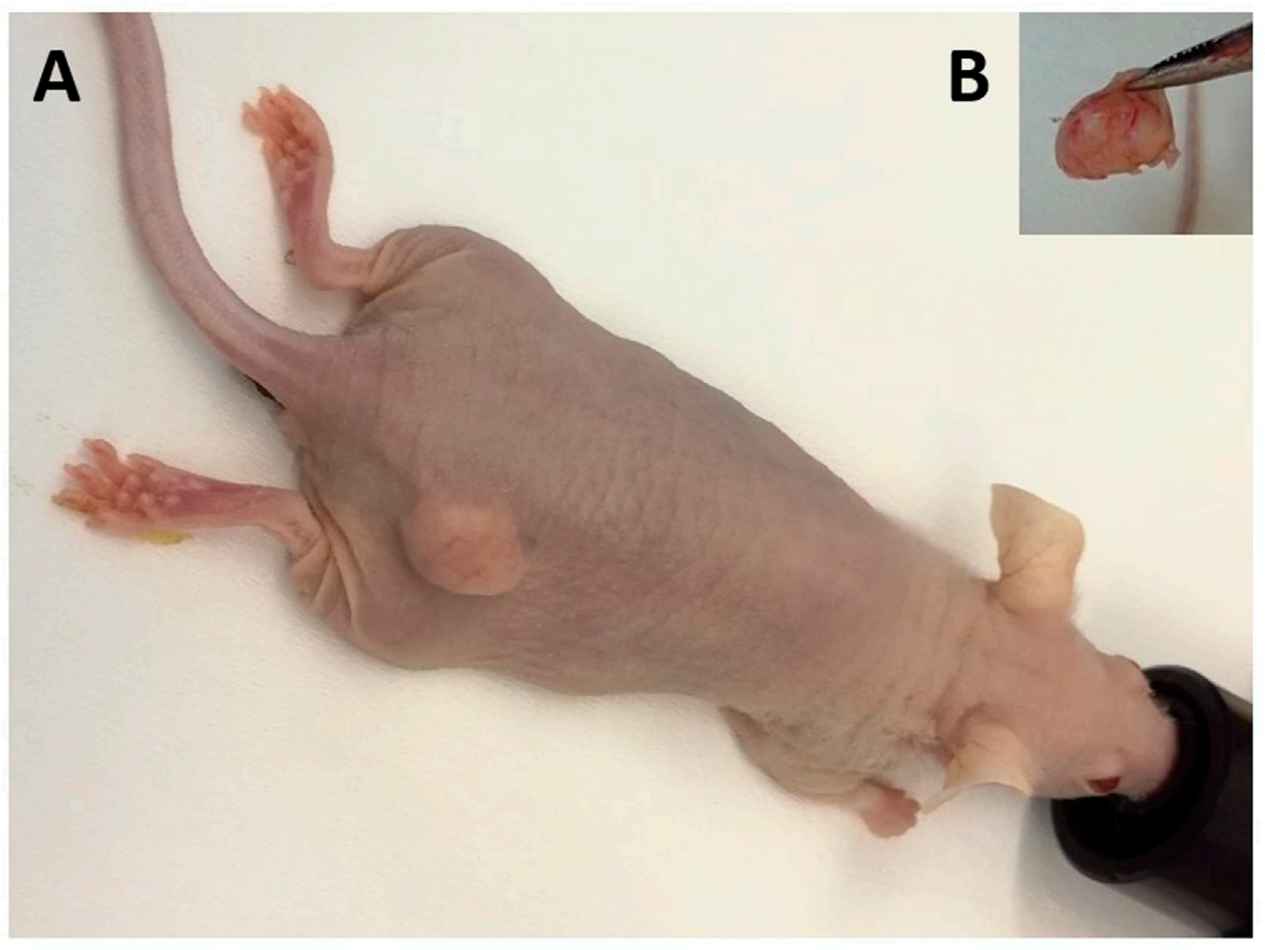
*In vivo* model of PTX resistant ovarian tumor. **(A)** Representative image of successful tumor growth after application of NCI/ADR-RES cells. **(B)** Representative image of resected tumors at the end of experiment.

Xenograft models of resistant ovarian cancer were prepared from the NCI/ADR-RES cell line. Application of PTX, third generation SB-Ts, and their combinations was initiated after tumor size reached ∼ 100 mm^3^. Examples of successful tumor growth and resected tumors are displayed on representative photo (Figures 7A,B). SB-T-121605 and SB-T-121606 were selected for the study of efficacy in mouse xenograft models as the most promising new SB-Ts on the basis of previous *in vitro* analyses.

#### 3.4.1 *In vivo* effect of the third generation SB-T-121605

**FIGURE 8:**
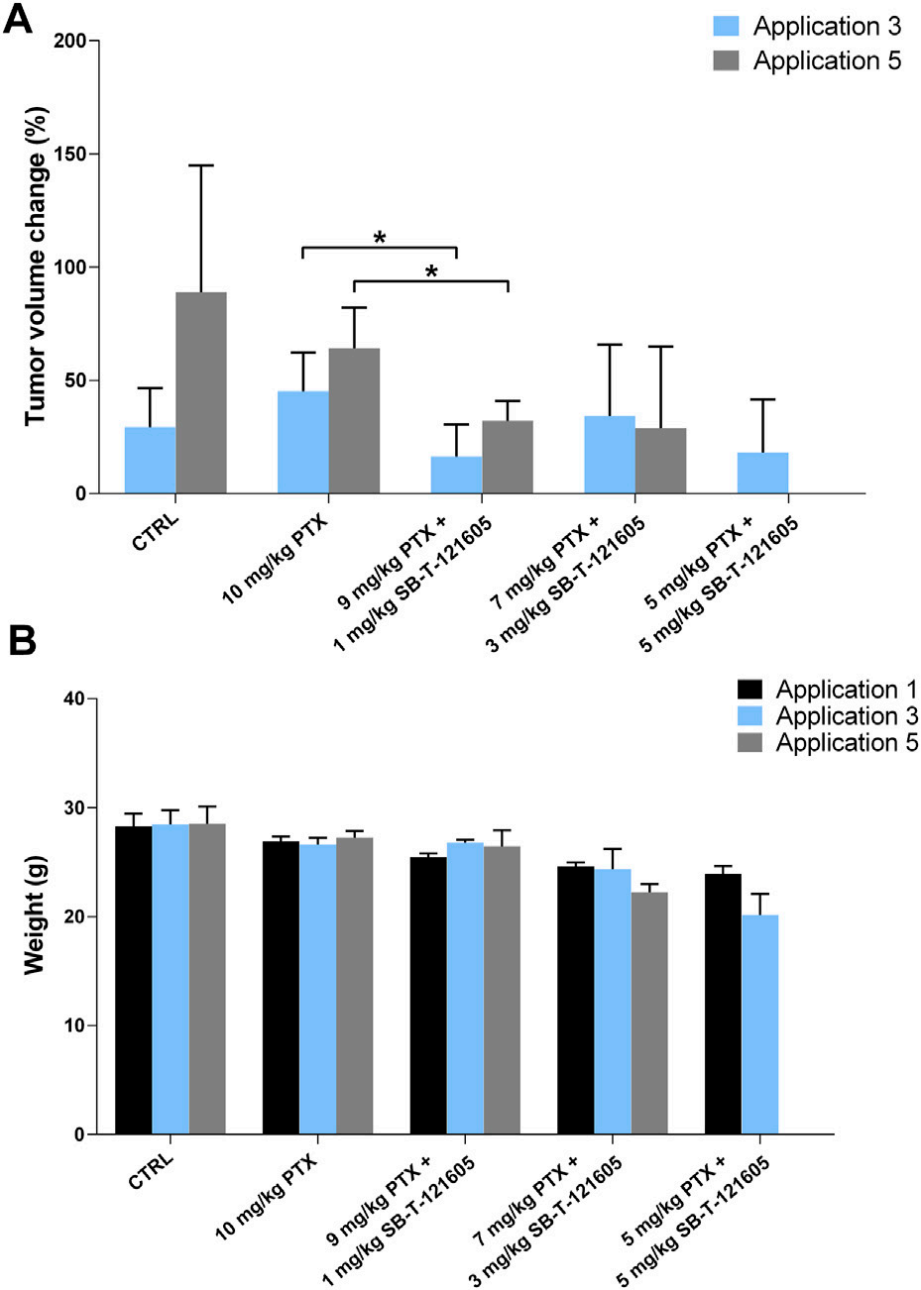
*In vivo* efficacy of SB-T-121605. **(A)** Changes of tumor volume (%) during experiment in comparison to situation before the first application. Efficacy was compared between control group, experimental group treated with 10 mg/kg PTX alone, and groups treated with combinations of a/9 mg/kg PTX with 1 mg/kg SB-T-121605, b/7 mg/kg PTX with 3 mg/kg SB-T-121605, and c/5 mg/kg PTX with 5 mg/kg SB-T-121605. **(B)** Changes of mouse weight (g) during the application in all examined experimental groups. *p-values by two-tailed Student´s t-test (*p ≤ 0.05).

**FIGURE 9:**
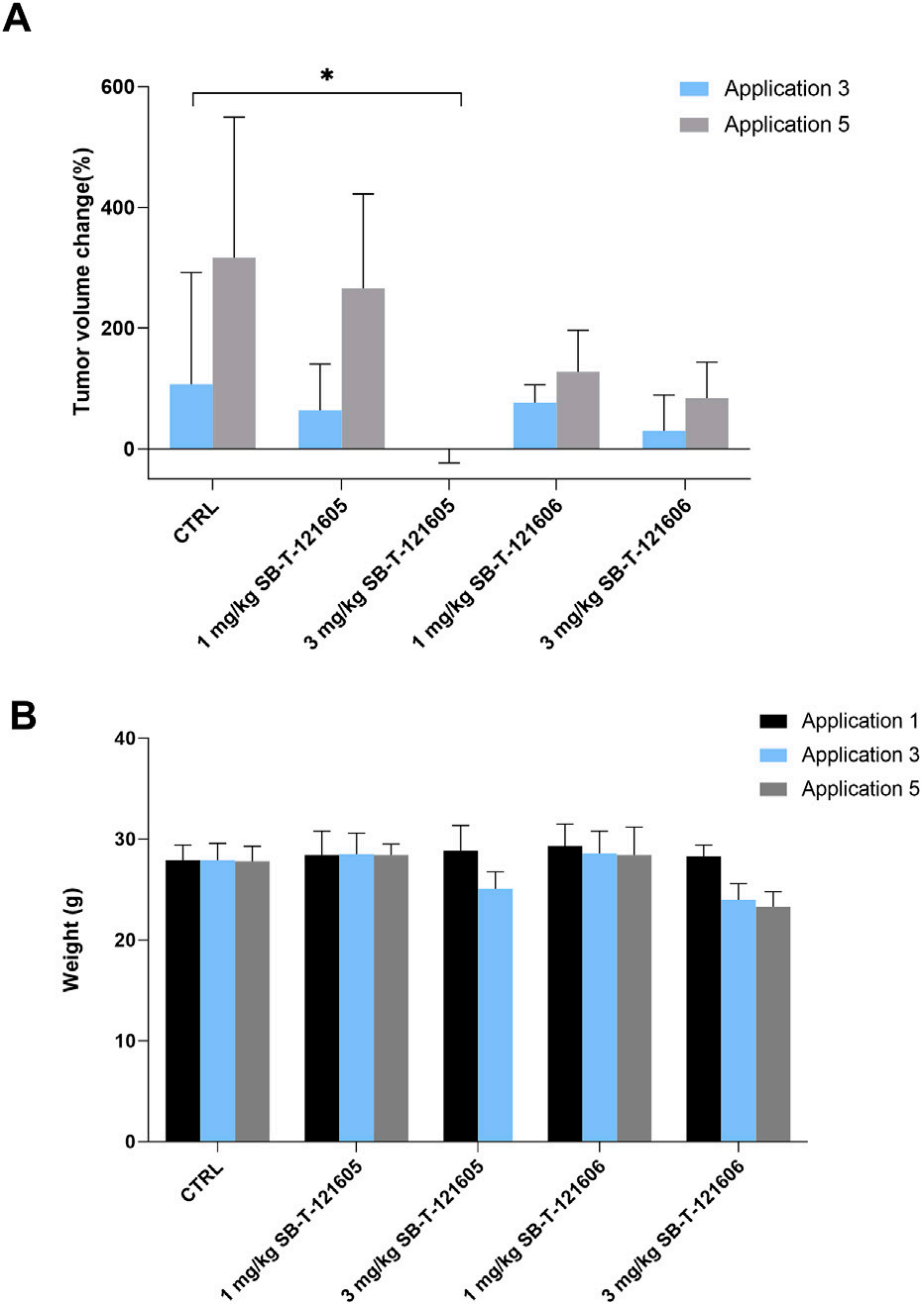
Comparison of *in vivo* efficacy of SB-T-121605 and SB-T-121606 administered alone in concentrations of 1 mg/kg and 3 mg/kg. **(A)** Comparison of proportional (%) change of tumor volume during experiment. **(B)** Changes of mouse weight (g) during the application in all examined experimental groups. *p-values by two-tailed Student’s t-test (*p ≤ 0.05).

Firstly, the efficacy of SB-T-121605 was tested in three different combinations with PTX; 1) 9 mg/kg PTX with 1 mg/kg SB-T-121605, 2) 7 mg/kg PTX with 3 mg/kg SB-T-121605, and 3) 5 mg/kg PTX with 5 mg/kg SB-T-121605. Our *in vitro* results suggest that very low levels of third generation SB-Ts are effective in comparison to higher doses, where systemic toxicity is observed. Combination regimens were also compared with 1 mg/kg and 3 mg/kg SB-T-121605 alone and 10 mg/kg PTX alone. Mice treated with five doses of PTX alone showed an increase of the tumor volume over the period of the PTX application, consistent with the control group treated with vehicle only (Figure 8A). A significant reduction of the tumor volume growth was observed in the group treated with the combination of 9 mg/kg PTX with 1 mg/kg of SB-T-121605 in comparison to the PTX group (*p* = 0.03 after third and *p* = 0.01 after fifth application). Combination of 7 mg/kg PTX with 3 mg/kg SB-T-121605 showed apparent slowdown of tumor volume growth after the fifth application (Figure 8A). Combination of 5 mg/kg PTX with 5 mg/kg SB-T-121605 also caused slowdown of tumor volume growth compared to PTX and other combination regimens (Figure 8A). However, combinations containing ≥ 3 mg/kg SB-T-121605 exerted higher toxicity in comparison to combinations with lower doses as can be seen from the decreasing mouse weight (Figure 8B). A very similar result was observed for the application of 3 mg/kg SB-T-121605 alone, which led to the reduction of tumor volume compared to control (*p* = 0.05) after the third application, but the systemic toxicity manifested by intestinal obstruction and overall physical wasting was too high. Group treated with a lower concentration of SB-T-121605 (1 mg/kg) showed manageable systemic toxicity for experimental mice, but no reduction in tumor volume (Figure 9). Taken together, the only regimen using SB-T-121605 without manifestation of serious systemic toxicity and simultaneous reduction of tumor volume was the combination of 9 mg/kg of PTX with 1 mg/kg SB-T-121605.

#### 3.4.2 *In vivo* effect of the third-generation SB-T-121606

**FIGURE 10:**
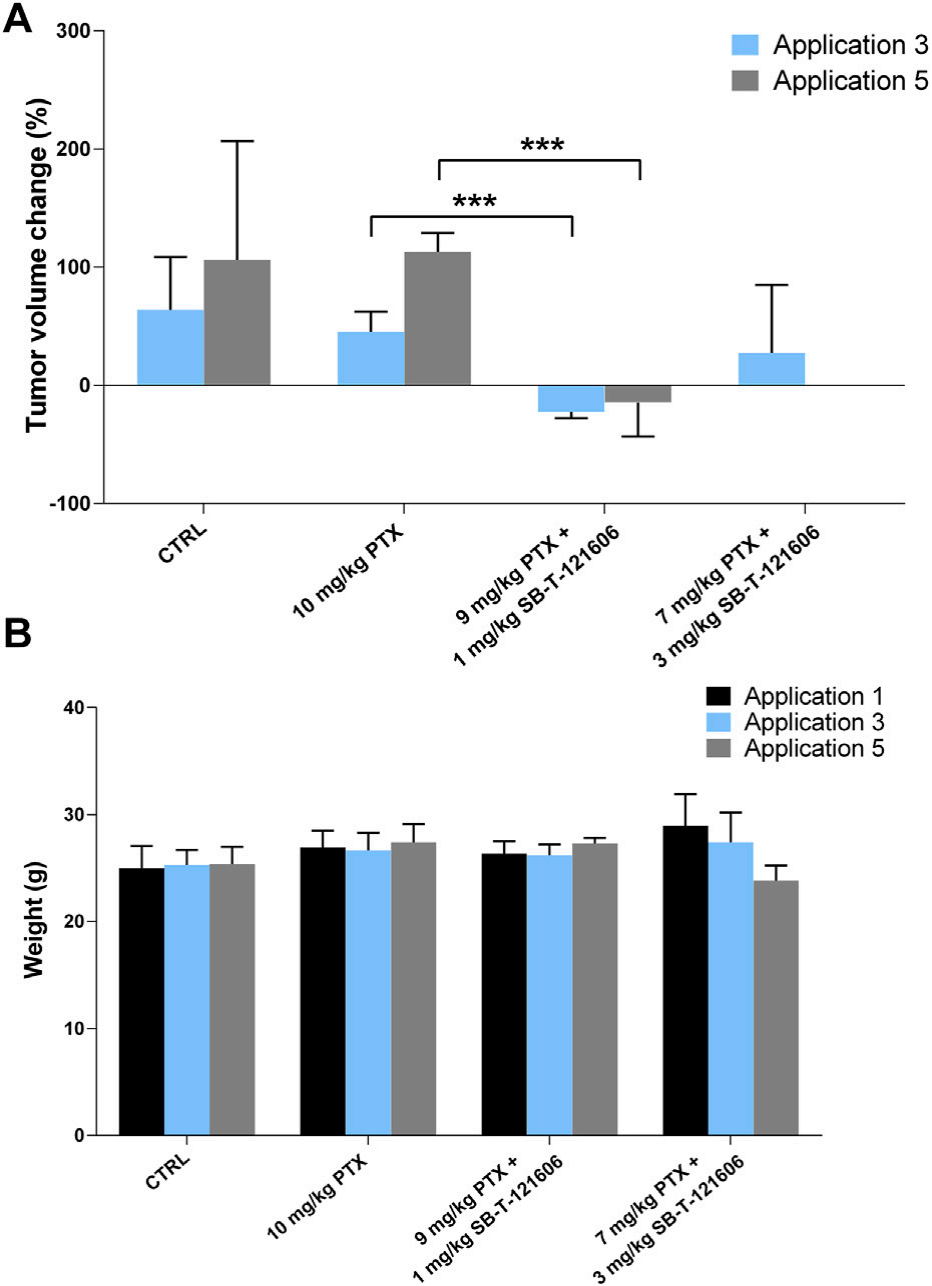
*In vivo* efficacy of SB-T-121606. **(A)** Changes of tumor volume (%) during experiment in comparison to tumor volume before the first application. Efficacy was compared between control group, experimental group treated with a/10 mg/kg PTX alone, b/combinations of 9 mg/kg PTX with 1 mg/kg SB-T-121606 and c/7 mg/kg PTX with 3 mg/kg SB-T-121606. **(B)** Changes of mouse weight (g) during the application in all examined experimental groups. *p-values by two-tailed Student’s t-test (***p ≤ 0.001).

Intrigued by the results of SB-T-121605, we tested the efficacy of SB-T-121606 in a five-dose scheme based on two combinations with PTX; 1) 9 mg/kg PTX with 1 mg/kg SB-T-121606 and 2) 7 mg/kg PTX with 3 mg/kg SB-T-121606 and compared effects to SB-T-121606 alone in two concentrations (3 mg/kg and 1 mg/kg), 10 mg/kg of PTX alone, and control group treated with vehicle only. The effect of SB-T-121606 on tumor volume is shown in Figure 10A. Both combinations of PTX with SB-T-121606 showed significant reduction of tumor volume during the treatment in comparison with control or PTX alone. Combination of 9 mg/kg PTX with 1 mg/kg SB-T-121606 showed a significant reduction of tumor volume growth in comparison with the PTX treated group after the third (*p* = 6.48E-05), fourth (*p* = 2.13E-05), and fifth applications (*p* = 0.0004). The combination of 7 mg/kg PTX with 3 mg/kg SB-T-121606 also reduced tumor volume growth after third application, although we observed large variability in the response of mice to the treatment in this group and high systemic toxicity (Figure 10A). The observed systemic toxicity of SB-T-121606 at concentrations of 3 mg/kg was analogous to the SB-T-121605 effect and manifested in the same way as in SB-T-121605, i.e. by intestinal obstruction and overall physical wasting (Figure 10B). On the other hand, both groups treated by SB-T-121606 alone (3 mg/kg and 1 mg/kg concentrations) suggested reduction in tumor volume growth during experiment, but the differences were not statistically significant (Figure 9). Again, the regimen combining 9 mg/kg of PTX with 1 mg/kg SB-T-121606 was the only effective regimen without serious systemic toxicity, but with reduction of tumor mass and consistency across the experiment.

#### 3.4.3 Comparison of the effective regimens of SB-T-121605 and SB-T-121606

**FIGURE 11:**
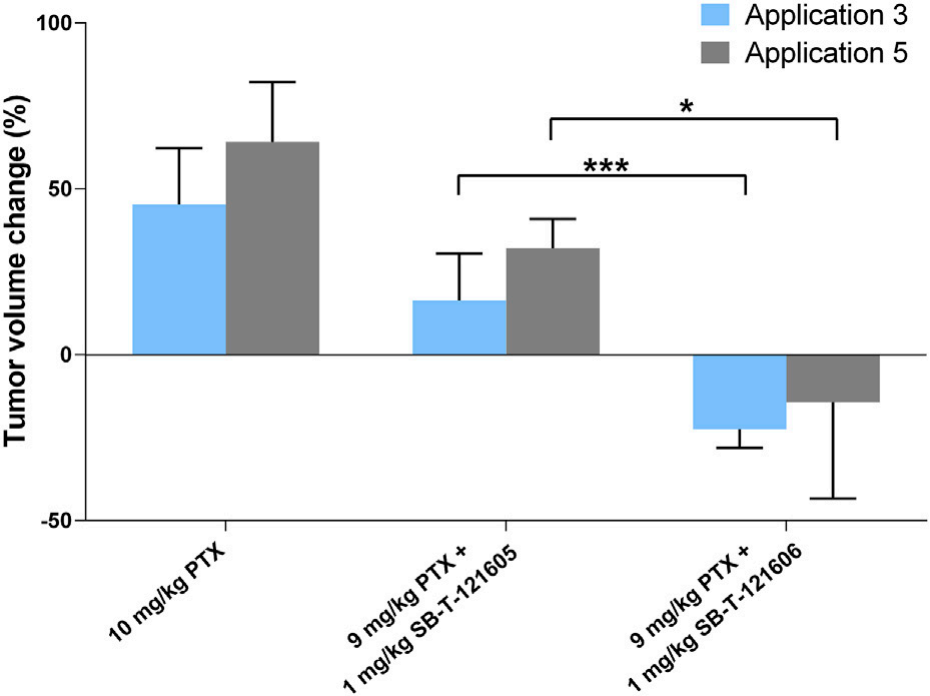
Comparison of *in vivo* efficacy of SB-T-121605 and SB-T-121606 combined with PTX. Comparison of proportional (%) change of tumor volume during experiment. Efficacy was compared between the groups treated with a/10 mg/kg PTX, b/combination of 9 mg/kg PTX + 1 mg/kg SB-T-121605 and c/combination of 9 mg/kg PTX + 1 mg/kg SB-T-121606. *p-values by two-tailed Student´s t-test (*p ≤ 0.05, ***p ≤ 0.001).

In order to identify the most effective combination regimen, we compared efficacy of PTX alone with regimens combining 9 mg/kg of PTX with 1 mg/kg SB-T-121605 or SB-T-121606. Combination of PTX with SB-T-121606 was significantly more effective in reducing the tumor volume than that with SB-T-121605 as shown in Figure 11. We consider the combination of 9 mg/kg PTX with 1 mg/kg of SB-T-121606 to be the most effective and stable regimen for treatment of resistant ovarian carcinomas in mouse xenograft models.

## 4 Discussion and conclusion

Resistance of tumor cells to conventional taxanes is a serious problem complicating successful therapy of many types of malignancies including ovarian carcinomas. Stony Brook taxanes of the second generation have been previously demonstrated to be highly effective in various types of sensitive and resistant breast, pancreatic, and ovarian carcinoma cell lines *in vitro* (Ehrlichova et al., 2005; Ehrlichová et al., 2012; Jelínek et al., 2015; Wang et al., 2020). However, their toxicity is relatively high and therefore structural modifications were suggested to improve this limitation. In the present study, we provide data on the efficacy of new third generation taxanes (SB-T-121402, SB-T-121605 and SB-T-121606) and their comparison to the second generation (SB-T-1214 and SB-T-1216) and clinically used PTX *in vitro* and *in vivo*. Furthermore, we report an innovative way of decreasing systemic toxicity, while maintaining high antitumor efficacy, of the third generation SB-Ts in a mouse xenograft model bearing the paclitaxel-resistant NCI-ADR/RES ovarian cancer cell model *in vivo*.

Firstly, our results show that third generation SB-Ts are more cytotoxic than second generation ones in the NCI/ADR-RES PTX-resistant cells *in vitro*. These results confirm findings of a study by Wang et al., analyzing a panel of new third generation SB-Ts bearing 3-CH3, 3-CF3O and 3-CHF2O groups at the C2-benzoate moiety of the taxane structure in drug-sensitive cancer cell lines (MCF7 and LCC6-WT) and drug-resistant ovarian, breast and colon cancer cell lines with multidrug resistance phenotype (NCI/ADR, LCC6-MDR and LDL-1) (Wang et al., 2020). Our study extended findings for highly effective third generation taxanes, SB-T-121605 and SB-T-121606, which induced cell cycle arrest in the G2/M phase much more effectively than PTX. Moreover, we found for the first time that intracellular content of the third generation taxanes inside the highly PTX-resistant NCI/ADR-RES cells was 10–15-times higher compared to PTX. In our previous studies, the NCI/ADR-RES cells accumulated 1.5-6.5 higher the amount of second generation taxanes than PTX (Ehrlichová et al., 2012). Thus, the novel third generation SB-Ts are more cytotoxic due to either higher uptake or lower efflux by tumor cells or both. Which one of these mechanisms is responsible is currently under investigation, but with regard to the fact that the NCI/ADR-RES cells overexpress ABCB1/P-glycoprotein (Ehrlichova et al., 2005), our results support the notion that third generation SB-Ts share lower affinity to this drug efflux transporter.

The third generation SB-Ts have different functional groups in the side chains (Figure 1) compared to the second generation SB-T-1214 and SB-T1216, which probably play a role in their overall cytotoxicity. Namely the modifications of the C2 benzoate moiety of structures SB-T-1214 and SB-T-1216 by the CH3, OCF3 or OCF2H functional groups seem relevant (Wang et al., 2020). The most cytotoxic taxanes found by our study, SB-T-121605 and SB-T-121606, have OCF3 and OCF2H groups at the C2 benzoate moiety of the SB-T-1216 parental structure. Molecular modeling of the third generation SB-Ts revealed considerably favorable van der Waals interactions of OCF3 and OCF2H groups with hydrophobic amino acid residues (Leu230 and Leu275) in the binding site and their high cell permeability, which was slightly higher for OCF2H than for the OCF3 analog (Wang et al., 2020). Furthermore, introduction of to OCF3 and OCF2H groups into drug candidates leads to improvement of their metabolic stability, membrane permeability and pharmacokinetic profile (Ojima et al., 2017; Meanwell, 2018). Collectively, these findings suggest that taxane structures with the above-mentioned functional groups may affect the metabolism by cytochromes P450 and binding to transporters involved in their translocation across cell membranes. Due to the above reasons and based on our *in vitro* and *in vivo* findings, we suggest that third generation SB-Ts may be used in lower concentrations than second generation SB-Ts.

Consequently, we further studied the effects of SB-T-121605 and SB-T-121606 *in vivo* using mice xenografts derived from PTX-resistant NCI/ADR-RES cells. We previously reported compelling data on the efficacy of fluorinated taxanes SB-T-12854 and SB-T-1214 in rat lymphomas, although this effect was accompanied by a relatively high systemic toxicity (Otová et al., 2012). In this study, both third generation SB-Ts administered alone were effective in resistant ovarian tumor growth suppression *in vivo*. However, doses of SB-Ts above 3 mg/kg were accompanied by considerable intestinal toxicity and overall physical wasting of mice. SB-Ts doses of 1 mg/kg showed manageable toxicity profile in mice, but no significant efficacy towards tumor volume reduction. To eliminate this toxicity profile and enhance efficacy, we suggest for the first time combinations of a low dose of the third generation SB-Ts with a higher dose of PTX. The combination regimens of 1 mg/kg of SB-T with 9 mg/kg of PTX proved to be highly effective in tumor growth suppression and even induced significant regression. Furthermore, SB-T-121606 seemed to be more potent than the SB-T-121605 derivative. Apparently, the observed high antitumor efficacy of SB-Ts combined with PTX and accompanied by the absence of signs of systemic toxicity demonstrate a novel option in the exploration of therapeutic regimens against resistant phenotypes in experimental oncology. Although no data is available about the third generation, our previous results on the second generation (Gut et al., 2006) suggested that liver cytochromes P450 are capable to metabolize SB-Ts and thus considerably influence the pharmacokinetics and pharmacodynamics. Follow up studies should answer the question of efficacy, pharmacokinetics, and pharmacodynamics of third generation SB-Ts.

The observed toxicity of SB-T-121605 and SB-T-121606 at higher doses represents a serious limitation of their use in future research. On the other hand, we demonstrate that combination regimens of third generation SB-Ts with PTX at considerably lower doses overcome this. In addition, third generation taxanes SB-T-121605 and SB-T-121606 would be potentially excellent payloads in tumor-targeting folate- or biotin-drug conjugates. Folate-drug conjugates are effective *via* the folate receptor (FR), which is highly expressed in some tumors in comparison to most healthy tissues (Xia and Low, 2010), and which has been widely recognized as an attractive target for tumor-selective delivery of cytotoxic agents (Seitz et al., 2015). Seitz et al. (2015) designed and constructed a folate conjugate of the highly potent, but toxic SB-T-1214 by exploiting bioorthogonal Cu-free ‘click’ chemistry. This technology could be readily used for the synthesis of folate-third-generation taxane conjugates (Seitz et al., 2015). Similarly, biotin receptor (BR) was found to be overexpressed even more than the FR in a number of cancer cell lines and has emerged as a promising molecular target for tumor-targeted drug delivery systems (Chen et al., 2010; Vineberg et al., 2015). Biotin-linker-taxoid SB-T-1214 conjugates were effective in human breast cancer cells, murine ovarian, and lymphocytic leukemia cells overexpressing biotin receptors. On the other hand, they were benign against normal cells (BR-) at the drug concentration used for chemotherapy (Vineberg et al., 2015). Nanoemulsion of the second generation SB-T-1214 (NE-nDHA-SBT-1214) represents another promising formulation which has shown significant activity against prostate CD133high/CD44+/high tumor-initiating cells both *in vitro* and *in vivo* (Ahmad et al., 2017). Very recently, another third generation SB-T, 3′-difluorivinyltaxoid (DFV-taxoid), also called SB-T-1285406, promoted the enhancement of tubulin polymerization in comparison to PTX in a faster manner. This is another potential mechanism behind the improved efficacy of SB-Ts (Wang et al., 2022). Thus, besides the newly established combination regimens presented in the present study, other options are being developed for optimizing the efficacy and managing side effects of treatments of resistant tumors.

Additionally, *in vivo* treatment of mouse models with SB-Ts offers the chance to address the role of changes of gene expression profile changes in tumors under therapeutic pressure and its potential for monitoring therapeutic efficacy of new drugs and regimens. In our previous studies, we deciphered pathway-specific effects of the second generation SB-Ts by analysis of changes in gene expression. Particularly, the treatment with second generation SB-Ts 1214 or 1216 led to the suppression of *ABCB1* gene expression and changes in the Hedgehog and KRAS signaling pathways’ expression profiles (Otová et al., 2012; Mohelnikova-Duchonova et al., 2017; Oliverius et al., 2019). The combined regimen composed of PTX with both third generation taxanes, used in the present study, caused downregulation of CPS1 in the PTX-resistant NCI/ADR-RES-derived mouse xenograft model *in vivo*. Furthermore, *CPS1* overexpression was also associated with poor survival of patients with epithelial ovarian carcinoma (Seborova et al., 2022). We therefore assume that a detailed study of effects of taxanes and their combinations on the gene expression profile (and potentially also epigenetic factors) may help decipher not only the mechanisms behind their antitumor activity, but also reveal new candidate biomarkers and/or targets enabling personalized therapy of solid tumors.

In summary, this study examined *in vitro* and *in vivo* efficacy of novel third-generation taxanes in comparison to the clinical mainstay, PTX. We demonstrated remarkably high potency of the third-generation taxanes, SB-T-121605 and SB-T-121606, in a highly PTX-resistant ovarian carcinoma model *in vitro*. The high efficacy of SB-T-121605 and SB-T-121606 has been confirmed for the first time *in vivo*—in a drug-resistant ovarian carcinoma mouse xenograft model. Here, it is particularly noteworthy that a low dose combination of SB-Ts with PTX (1 mg/kg of SB-T and 9 mg/kg of PTX) exhibited considerably high efficacy without appreciable systemic toxicity. The third generation SB-Ts are highly promising drug candidates for further research on the effective treatment of resistant ovarian carcinomas and eventually other aggressive tumors, e.g. pancreatic carcinomas. Our data also indicate that careful optimization of the dose and combination therapy is beneficial in mitigating undesirable systemic toxicity and adverse effects of novel drug candidates.

## Data Availability

Data are available upon reasonable request to the corresponding author.
